# Impacts of climate change on current and future invasion of *Prosopis juliflora* in Ethiopia: environmental and socio-economic implications

**DOI:** 10.1016/j.heliyon.2020.e04596

**Published:** 2020-08-01

**Authors:** Dejene W. Sintayehu, Gemedo Dalle, Arbo F. Bobasa

**Affiliations:** a College of Agriculture and Environmental Sciences, Haramaya University, Dire Dawa, Ethiopia; b Center for Environmental Science, College of Natural and Computational Sciences, Addis Ababa University, Ethiopia

**Keywords:** Climate suitability, Conservation planning, Environmental and economical impact, Ethiopia, Management of invasive species, Species distribution models, Climatology, Ecology, Environmental impact assessment, Geography, Plant biology, Environmental science

## Abstract

*Prosopis juliflora* is a serious invader, causing great ecological and economic damage in Ethiopia. Thus, it is imperative to examine potential invasion dynamics of *P. juliflora* at national level under climate change scenario to better influence decision making processes on the management of this invasive species. We derived a consensus model from five modeling approaches to examine the current and future (2050 and 2070) climatic suitability for *P. juliflora* under two climate scenarios (RCP4.5 and RCP8.5) in Ethiopia. Under the current climatic scenario, 94.8% of the country was non-suitable for *P. juliflora* establishment and invasion while 0.4% (4.56 million ha) was highly suitable. In 2050, highly suitable area for *P. juliflora* is expected to increase by 55.6% and 63.6%, while moderately suitable area is projected to increase by 33.3% and 42.9% under RCP4.5 and RCP8.5 climate scenarios, respectively. Compared to the current climatic condition, in 2070, highly suitable area for the species is projected to increase by 73.3% (3.43 million ha) and 80.0% (3.65 million ha) under RCP4.5 and RCP8.5 scenario, respectively. With the current cover, this invasive species had already caused significant impact on rangelands in many parts of the country. Its further expansion would worsen the problem, leading to great environmental and economic damage, thereby threatening the livelihood of the community. Negative environmental and economical impacts caused by the species will be high if preventive and effective management measures are not earnestly taken, and it becomes one of the major challenges for the 21^st^ century pastoralism and their livelihoods. We recommend a national effort be organized towards combating *P. juliflora* expansion to new areas, especially in regions and protected area predicted as frontiers of potential expansion.

## 1 Introduction

Biological invasions are one of the main drivers for the loss of biodiversity. They have been linked with the extinctions about 60% of species during the last century (Bellard et al., 2018). They also seriously affect ecosystem services and economic growth (Simberloff et al., 2013). Because of climate change and habitat fragmentation, the problem caused by biological invasions is expected to increase (Beaury et al., 2020; Hulme, 2009). Climate changes can facilitate introduction, establishment and spread of invasive species (Diez et al., 2012; Qin et al., 2016; Shiferaw et al., 2019a; Wakie et al., 2014), and subsequently have a significant negative impact on the environment. For instance, some invasive species are shifting their geographic distribution towards high altitude as the climate warms (Bradley et al., 2010; Shrestha et al., 2018), and new invasive species are adding to those currently being successfully controlled. Additionally, climate change stresses native ecosystems (Bradley et al., 2010; Corlett and Westcott, 2013) and increases disturbances through climate extremes (Diez et al., 2012), potentially provide new opportunities for establishment and spread on invasive species. Thus, it is crucial to examine the relationship between climate change and invasive species to design appropriate management strategies. Moreover, information is needed in order to design effective invasive species management that also accounts for climate change.

*Prosopis juliflora* was introduced in Africa for different purpose. For instance, it was first introduced in Sudan in 1917 with the aim to support combating desertification and provision of fuel-wood (Hoshino et al., 2012). In the 1980s, this species was brought to Lake Baringo of Kenya to similarly help address the issue of fuel wood shortage (Mbaabu et al., 2019). Similarly, *P. juliflora* was introduced in Ethiopia in the late 1970s to combat desertification. Now, the species is listed among the world's ten worst invasive weeds (Shrestha et al., 2018) and emerged as a significant threat to Africa's ecological landscapes (Wakie et al., 2014). Nationally, the species is one of the worst invasive species, threatening the environment in arid and semi-arid ecosystem of Africa, and affect the livelihood of pastoral and agro-pastoral society (Bekele et al., 2018; Mbaabu et al., 2019; Shackleton et al., 2015; Zeray et al., 2017). Recent studies showed that rate of *P. juliflora* invasion is increasing significantly, suppressing native plant species (Ayanu et al., 2014; Haregeweyn et al., 2013; Shiferaw et al., 2019c, Shiferaw et al., 2019d). *P. juliflora* creates a favorable environment for mosquito breeding, blocks access to key grazing and watering points and offers shelter for lions (*Panthera leo*) and other wild predators (Mehari, 2015). In Ethiopia, pastoralists named it the “Devil Tree” and the “AIDS” for livestock. Despite its negative impact, efforts made to control expansion of this invasive alien species in Africa have not been successful.

The total area covered by *P. juliflora* in Afar Region was estimated to be about 1.17million ha and the rate of invasion has been increased at annual rates of 31,127 ha (Shiferaw et al., 2019a). This invasive species has covered more than 12,000 ha in Dire Dawa City Administration (EBI, 2015; Haji and Mohammed, 2013). Furthermore, most of the grass and bush lands in the lowland areas of Oromia Regional State have been invaded by this invasive alien species (EBI, 2015). Studies also showed that *P. juliflora* has already threatened not only rangeland but also agricultural land and other vegetation types in arid and semi-arid ecosystems in several other parts of Ethiopia (FAO, 2006; Mohammed et al., 2018; Shiferaw et al., 2018). The recent expansion of *P. juliflora* to protected areas were also very high for instance Awash National Parks and Allaidege Wildlife Reserves and has become a national and international concern as biodiversity in these protected areas (both plants and animals) have been negatively affected (Mehari, 2015). Climate change profoundly influences the geographic distribution of *P. juliflora* (Heshmati et al., 2019; Shiferaw et al., 2018, 2019a). In Ethiopia, temperature has increased by 0.37 °C per decade since 1990s (EEA, 2008) and predicted to increase between 0.9 to 1.1 °C by 2030, 1.7–2.1 °C by 2050 and 2.7–3.4 °C by 2080 compared to the 1961–1990 average (Conway and Schipper, 2011). Annual precipitation is also expected to increase in many parts of eastern Africa including Ethiopia (Sintayehu, 2018). Climate change may provide an opportunity for establishment and geographical spread of *P. juliflora* and reduce habitat range for native species. Thus, the risk of invasion might be very high given the current rate of national climate warming. Given the negative impacts of the species on the environment, economy, and society, it is crucial knowing the relationships between climate change and invasion of *P. juliflora* for early detection, and successful management of the species (Beaury et al., 2020).

Species distribution model is a useful tool for simulating the spatial distributions of species and provide an opportunity for early detection before they become widespread in new areas. Ecological niche modeling (ENM) of species distribution model is now widely used to computerize algorithms to predict the distributions of a species across a geographical space and time, based on observed distributions of a species as a function of environmental conditions (Araújo and New, 2007). Several studies in Ethiopia have focused on its impacts on land use, land cover and livelihoods (Bekele et al., 2018; Shiferaw et al., 2019a; Zeray et al., 2017), spatial coverage in small geographical area in particular in Afar Regional State (Shiferaw et al., 2019c, Shiferaw et al., 2019d; Wakie et al., 2014), and biodiversity loss (Mehari, 2015; Shiferaw et al., 2019c, Shiferaw et al., 2019d; Zeray et al., 2017) in small geographical area in particular in Afar Regional State. However, a dearth of information on spatial distribution at national scale in Ethiopia and the relations between *P. juliflora* expansion and climate change dynamics as projected have been explored at a national level, in spite of the fact that this invasive plant species is rapidly expanding at the rate of 31,127 ha annually in Afar Regional State (Shiferaw et al., 2019a) and causing biodiversity loss (Shiferaw et al., 2019c, Shiferaw et al., 2019d; Wakie et al., 2014; Witt et al., 2018).

Our understanding of future trends in distribution of *P. juliflora* in Ethiopia is limited This study aimed at filling this national gap to positively influence decision making processes for control and proper management of the species. In this regard, we examined the current and future spatial distribution and climatic suitability for *P. juliflora* establishment and potential invasion dynamics at national level under climate change. Our specific objectives were to (a) assess the relative importance of environmental variables for *P. juliflora* establishment and invasion; (b) map the current and future habitat suitability for *P. juliflora* under different projections of climate and land use change; and (c) assess habitat change for the future habitat suitability projections for *P. juliflora* in Ethiopia. Defining climatic suitability for *P. juliflora* at national level is critical for early detection and support the national ongoing invasive species control and management strategies.

## 2 Materials and methods

### 2.1 Study area

**Figure 1:**
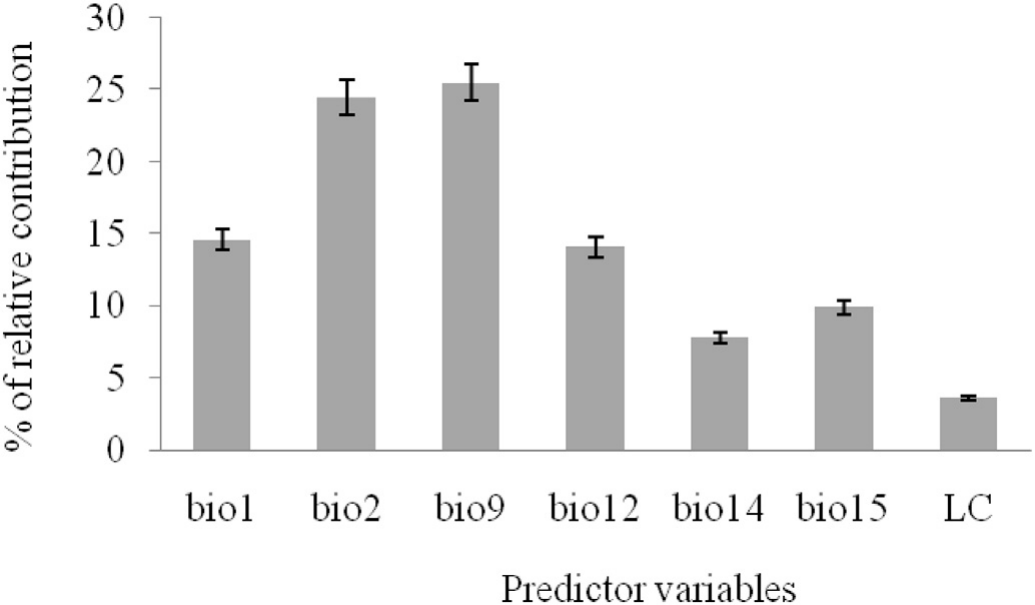
The mean relative importance of predictor variable (bio1 = annual mean temperature, bio2 = diurnal range, bio9 = mean temperature of driest quarter, bio12 = annual precipitation, bio14 = precipitation of driest month, bio15 = precipitation seasonality, LC = Land cover).

Ethiopia is located in the Horn of Africa within 3–15°N and 33–48°E (please see SI-Informations.doc SI-Figure 1), bordered with Kenya to the south, Somalia to the south and east, Djibouti to the east, Eritrea to the north and Sudan to the north west and South Sudan to the west. The country covers about 1.14 million km^2^, which is characterized high and rugged plateaus and the peripheral arid and semi-arid lowlands. The elevations of the country range from 126 m below the sea level in the Danakil Depression to 4620 m above the sea level on Mount Ras Dashen (CSA, 2016).

### *2.2* *P. juliflora* occurrence data

*Prosopis juliflora* presences records used to conduct ecological niche modeling were obtained from a number of sources. Occurrence points were also retrieved from the Global Biodiversity Information Facility (GBIF; www.gbif.org/ accessed April 10 2020), reports (FAO, 2006) and recent studies (Mohammed et al., 2018; Shiferaw et al., 2018; Wakie et al., 2014; Zeray et al., 2017). All points were mapped using ArcGIS 10.8 for visual observation and check spatial accuracy. Duplicate records were checked and removed. A total of 662 presence records were used to build the models. Again, 500 pseudo-absence points were generated by means of random sampling. To avoid the influence of false absences, we checked and removed points that were closer than 10 km to species presence point following the method of Eckert et al. (2020).

### 2.3 Environmental predictors

A total of 19 bioclimatic variables at 30-arc-sec resolution were obtained from WorldClim version 2 (http://worldclim.org/version2, accessed on April 3 2020). For future projections, we used an improved fifth version of the atmosphere-ocean General Circulation Model (GCM), from the Model for Interdisciplinary Research on Climate (MIROC), downloadable from the Worldclim website. MIROC5, which was used for the Intergovernmental Panel on Climate Change (IPCC) Fifth Assessment Report (AR5), is an important tool that significantly improved the descriptions of climatological features for better performance of climate change simulations. Currently, it is not clear which future climate change scenario provides the best predictions for invasive species (Hayes and Piaggio, 2018), thus we used two Representative Concentration Pathways RCP4.5 and RCP8.5 for the greenhouse gas concentration trajectories of 2050 and 2070. RCP 4.5 represents a stabilization scenario while RCP8.5 represents a worst case scenario and provides very high greenhouse gas emissions, atmospheric concentrations, air pollutant emissions and land use changes. Thus, we used both RCP4.5 and RCP8.5 climate change scenario in our analysis.

Bioclimatic variables that met three criteria were selected (Ren et al., 2020): those that (1) are statistically important in predicting *P. juliflora* presence data, (2) are biologically important for establishment and invasion of *P. juliflora*, and (3) do not display collinearity with other bioclimatic variables. We used Variance Inflation Factors (VIF) to detect collinearity among predictors, to minimize redundancy among the initial variable set. Using a stepwise procedure, we excluded all variables with VIF values larger than 3. Accordingly, three precipitation-related variables and three temperature-related variables, totaling six bioclimatic variables were used to build the final model in R statistical software (please see SI-Informations.doc SI-Table 1). Additionally, land cover was used to create distribution models for *P. juliflora*.

### 2.4 Species distribution modeling

Several algorithms are available to conduct ecological niche modeling and their prediction performances are different (Elith et al., 2006). A single algorithm does not give the best predictive accuracy in SDM, therefore an ensemble of multiple algorithms is recommended to produce better accuracy (Araújo and New, 2007). Our species distribution modelling approach was thus based on five modelling algorithms analyzed under the SDM package in R statistical software: (1) Generalized Linear Model (GLM), (2) Support Vector Machine (SVM), (3) a random forest algorithm (RF), (4) boosted regression trees (BRT), and (5) Multivariate Adaptive Regression Splines (MARS). We merged five prediction models into an “ensemble” by averaging the models with a true skill statistic (TSS) higher than 0.75 to get a “consensus model” and to avoid the integration of weak models (Allouche et al., 2006). Since predictions of invasive species distributions can vary widely among modeling approaches, the consensus methods was used to reduce the predictive uncertainty of single-models. In this regard, ensemble forecasting can enable a more robust model and overcome the uncertainties derived from each individual model (Araújo and New, 2007). The presence and pseudo-absence data were divided into two sets: 70% of the data were used for training the models while 30% were used for evaluating the model accuracy (Araújo and New, 2007). Finally, the areas of suitability changes for the year 2050 and 2070 were analyzed under four categories to identify the areas of no suitable, low suitable, moderate suitable and high suitable using ArcGIS 10.8.

### 2.5 Model performance evaluation

The overall performance of the model was assessed based on the threshold-independent area under the receiver operating characteristic curve (AUC) (Liu et al., 2005) and the threshold-dependent true skill statistic (TSS) (Allouche et al., 2006). The AUC values ranges between 0 and 1, whereas values of >0.9 are considered to be high performance, 0.7 to 0.9 moderate, 0.5 to 0.7 low and <0.5 no better than random (Phillips et al., 2006). The values of TSS indicators range from -1 to 1, where below 0 indicates bad and 1 high model performance. Models with a performance of <0.5 were discarded based on Allouche et al. (2006). Model performance was considered as ‘good’ only if both measures (AUC and TSS) were fulfilled. All analyses were conducted using the SDM package for R statistical software v.3.6.3 (R Core Team, 2017).

### 2.6 Change assessment

We assessed changes in suitable habitat between current and future (2050 and 2070) climate conditions in both RCP4.5 and RCP8.5 climate change scenario, mainly by identifying climate suitability (areas where suitable habitat was predicted in the present and future) and gain or loss assessment. The areas of suitability changes for the current and future (2050 and 2070) were analyzed under four categories to identify the areas of no suitable, low suitable, moderate suitable and high suitable using ArcGIS 10.8. We used two indicators to examine the role of climate change on the invasion of *P. juliflora*: (1) the change in the percentage of non suitable area (AC); (2) the percentage lost or gain areas by the 2050 and 2070 (CH). Indicators were calculated as:

$$AC=\frac{Af-Ac}{Ac}x100\%$$

$$CH=\frac{Af-Ac}{Af}x100\%$$

where Af is the predicted area of suitable habitat for *P. juliflora* in the future; and Ac is the predicted area of non suitable habitat under current conditions.

## 3 Results

### 3.1 Species distribution models of *P. juliflora*

**Figure 2:**
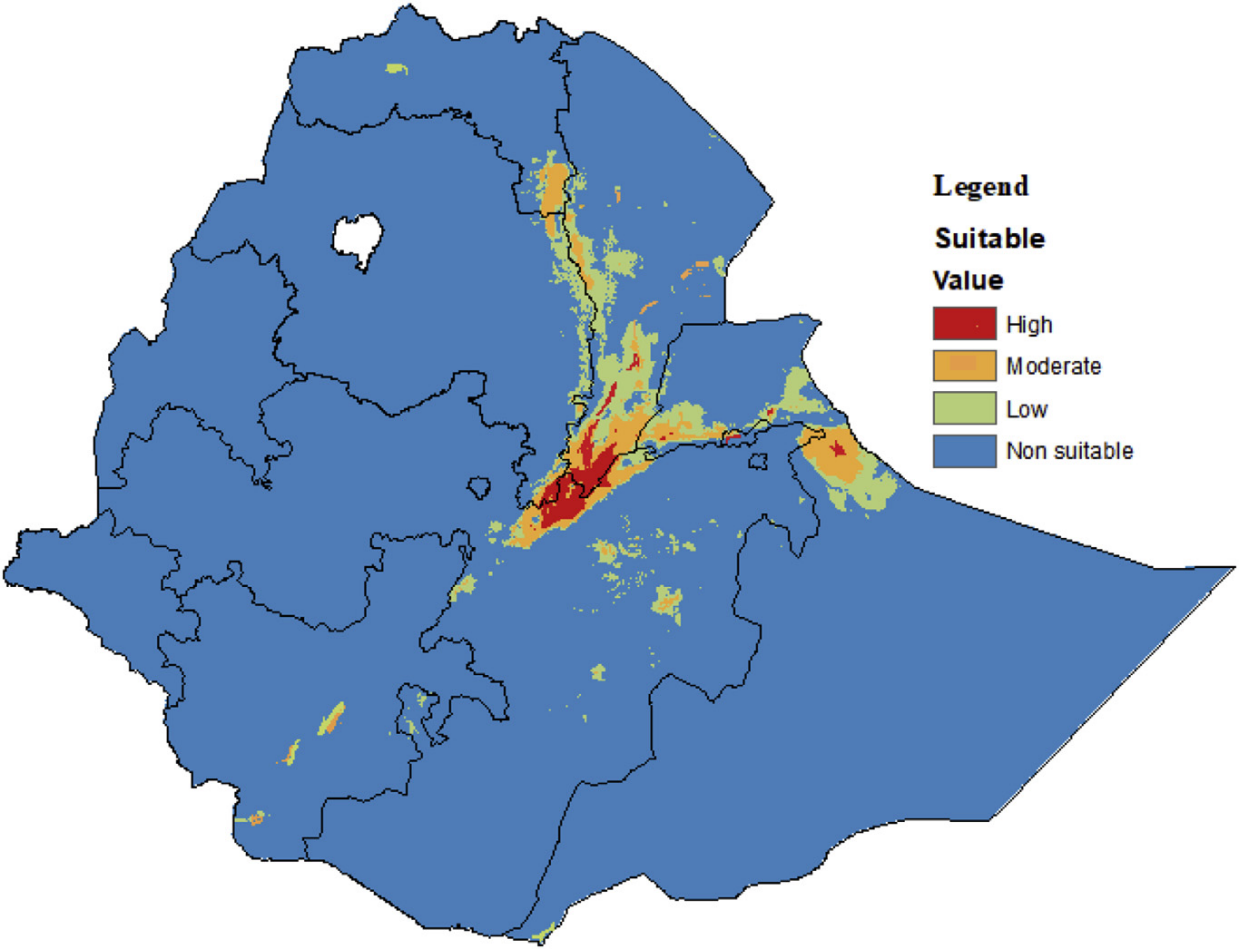
Habitat suitability for *P. juliflora* under current climatic conditions. Blue to red colors shows the gradient of suitability from low to high.

Based on the AUC and TSS assessments, the predictive performances of the models were very good (please see SI-Informations.doc SI-Figure 2). The mean AUC values of the five models ranged from 0.87 (lowest) from BRT to 0.96 (highest) from GLM with an overall average of 0.92. The mean TSS values of the models were 0.88. Among the five SDM models, RF and SVM received the highest (0.95) and the lowest (0.91) sensitivity, respectively.

The relative contribution of each predictor variables to individual models were analyzed (Figure 1). Of all the predictors' variables, mean temperature of driest quarter (bio9) was found to be the most contributing variable affecting the distribution of *P. juliflora*, followed by diurnal range (bio2) and annual mean temperature (bio1) by explaining 24.5%, 14.6%, and 14.1% of the variation in the model, respectively (Figure 1). The contribution of land covers was 3.7%.

### 3.2 Current predicted *P. juliflora* distributions

The predicted model showed that 94.8% of the country is non suitable for *P. juliflora* under the current climatic conditions while 0.4% is highly suitable (Table 1). We find that additional 3.2% and 1.6% of Ethiopia has a low and moderate suitability for *P. juliflora*, respectively.

**Table 1:** Percentage of current and future (2050 and 2070) climatic suitability class for *P. juliflora* in Africa under RCP4.5 and RCP8.5 climate change scenario.

Decades	Scenarios	Total suitability (%)			
		Not suitable	Low	Moderate	High
Current	-	94.8	3.2	1.6	0.4
2050	RCP4.5	92.6	4.1	2.4	0.9
	RCP8.5	90.9	5.2	2.8	1.1
2070	RCP4.5	89.6	5.6	3.3	1.5
	RCP8.5	88.6	5.7	3.7	2

*P. juliflora* has a geographically narrow distribution in the country under the current climatic condition covering significant parts of Afar Region and adjacent Amhara, Oromia and Tigray Regions. Its distribution is especially widespread within the north eastern part of the country but also extends to east including Dire Dawa city Administration and Somali Region (Figure 2).

### 3.3 Future predicted *P. juliflora* distributions

Compared to the current distribution, by 2050, the total area of highly suitable area for *P. juliflora* under RCP4.5 and RCP8.5 will gradually increase to 0.9% and 1.1%, respectively. The total area of the unsuitability for *P. juliflora* will decrease by 2.4% and 4.3% under RCP4.5 and RCP8.5, respectively, whereas high suitability for the species will increase by 55.6% and 63.6% respectively (Table 2). Under similar scenario RCP4.5 and RCP8.5 in 2050s, the total moderate suitable area is projected to increase to 33.3% and 49.9% under RCP4.5 and RCP8.5 scenario, respectively. Overall, areas considered with low suitability in the country will increase to 21.9% and 35.5% under RCP4.5 and RCP8.5 scenarios. Compared to the current climatic condition, in 2070, highly suitable climate for the species is projected to increase by 73.3% and 80.0% under RCP4.5 and RCP8.5 scenario, respectively. Moreover, moderately suitable area will increase by 51.5% and 56.8% under RCP4.5 and RCP8.5 climate scenario in 2070, respectively. In the same period, the total non suitable area for *P. juliflora* under RCP4.5 and RCP8.5 scenario is expected to decrease by 5.8% and 7.0%, respectively (Table 2).

**Table 2:** Percentage of change (gain or loss) of suitability for *P. juliflora* under current and future (2050 and 2070) climate change in Ethiopia under RCP4.5 and RCP8.5 climate change scenario.

Decades	Scenarios	Change (%) compared to the current suitability			
		Not suitable	Low	Moderate	High
Current	-	-	-	-	-
2050	RCP4.5	-2.4	21.9	33.3	55.6
	RCP8.5	-4.3	38.5	42.9	63.6
2070	RCP4.5	-5.8	42.9	51.5	73.3
	RCP8.5	-7.0	43.8	56.8	80.0

**Figure 3:**
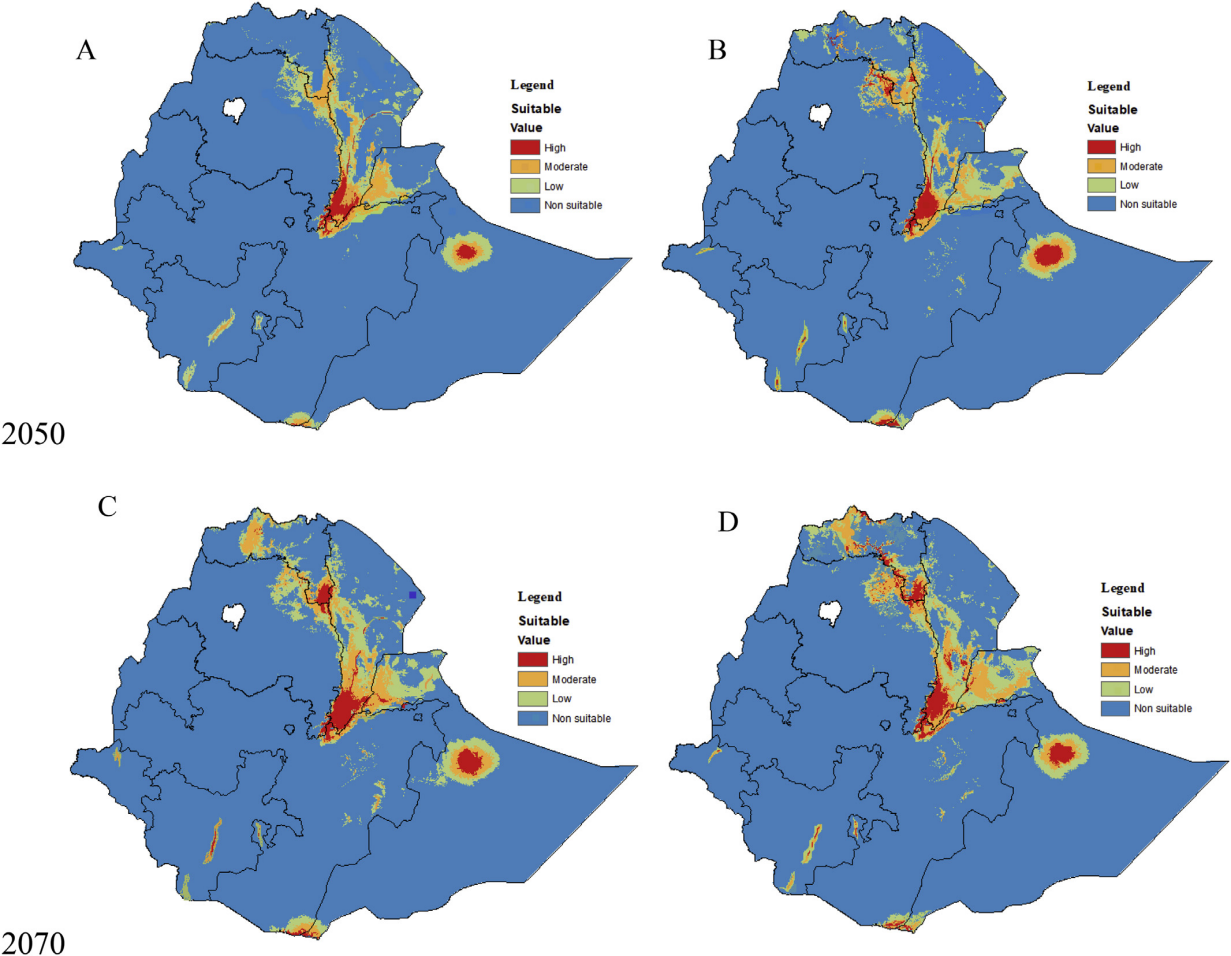
Future habitat suitability projections for *P. juliflora* by 2050 under RCP4.5 (A) and RCP8.5 (B), and by 2070 under RCP4.5 (C) and RCP8.5 (D) in Ethiopia. Blue to red colours illustrate gradients of habitat suitability from low to high.

The future model projections map revealed possible expansion in the potential distribution of *P. juliflora* (Figure 3). South Nation Nationalities and Gambella Regions currently unsuitable for *P. juliflora* colonization but projected to become suitable by 2050 and 2070 (Figure 3) included northern parts of Amhara and Tigray Regions, parts of several eastern part of Oromia Region and Dire Dawa City Administration. Similar probabilities shifts were also identified in fragmentary regions mostly adjacent to currently suitable areas.

### 3.4 Vulnerability assessment

The predicted model indicated that current non suitable habitat for *P. juliflora* would be vulnerable to be invaded by the species. Under future climate conditions, the non suitable habitat area of *P. juliflora* was vulnerable to losses of 2.3% and 4.1% under RCP4.5 and RCP 8.5 climate change scenario, respectively, by 2050. The percentage of vulnerability further increase by 5.5% and 6.5% under RCP4.5 and RCP 8.5 climate change scenario, respectively, by 2070 (Table 3).

**Table 3:** Projected *P. juliflora* suitable class vulnerability in Ethiopia.

Decades	Scenarios	Vulnerability (%) Compared to the current suitability			
		Not suitable	Low	Moderate	High
Current	-	-	-	-	-
2050	RCP4.5	-2.3	4.3	4.0	2.3
	RCP8.5	-4.1	9.6	5.9	3.3
2070	RCP4.5	-5.5	11.5	8.5	5.2
	RCP8.5	-6.5	11.9	10.4	7.5

### 3.5 Risk of protected areas for *P. juliflora*

According to the current climatic scenario, Awash National Park, Allaidege Wildlife Reserve and community conservation in Somali Regional State were identified as highly invaded by *P. juliflora* (please see SI-Informations.doc SI-Figure 3A). In the future high to moderate establishment and invasion of the species is predicted to occur in Kafta Sheraro National Park in the northern, Gambella National Park in the western, Nech Sar, Omo and Mago National Parks in the south western and Babile Elephant Sanctuary in the eastern part of the country (please see SI-Informations.doc SI-Figure 3B).

## 4 Discussion

Climate change facilitates and create opportunity for the establishment and spread of invasive species and also reduces the resilience capacity of native species (Hellmann et al., 2008). Recent studies have concluded that climate change will greatly affect invasive species distributions by causing expansions, shifts, or contractions in the species ranges (Corlett and Westcott, 2013; Hellmann et al., 2008; Sintayehu, 2018; Thomas et al., 2004) Similarly our ensemble model showed that climate change would significantly influence the establishment and distribution of *P. juliflora* in Ethiopia. The model predicted that there would be a gain in both the highly and moderately suitable habitats in the country particularly in the eastern, northwestern and southwestern part of the country. Under the current climatic condition, our suitability model is in agreement with the current distribution of the species (Mohammed et al., 2018; Shiferaw et al., 2018, 2019b; Wakie et al., 2014; Zeray et al., 2017).

*P. juliflora* is listed among the top ten worst invasive alien species in country (EBI, 2015). According to various studies (Felker et al., 2001; Getachew et al., 2012; Mbaabu et al., 2019; Shackleton et al., 2015) the species tends to inhibit regeneration of native species as it suppresses undergrowth. The leaves of *P. juliflora* contain various chemicals such as tannins, flavinoids, steroids, hydro-carbons, waxes and alkaloids which are known to have negative impacts on the germination and growth of other plant species (Felker et al., 2001; Getachew et al., 2012; Shiferaw et al., 2019c, Shiferaw et al., 2019d, 2018). The species forms intermingled and interwoven branches at its early stage of growth and prevents sunlight reaching to the under canopy vegetation, thereby negatively affects local biodiversity (Shiferaw et al., 2019c, Shiferaw et al., 2019d; Wakie et al., 2014). Furthermore, with its deep root system, it survives well in moisture stressed ecosystems over competing other species especially herbaceous species. Invasive species like *P. juliflora* have the inherent ability to tolerate wider environmental ranges or adapt to new environmental conditions (Kariyawasam and Kumar, 2019; Qin et al., 2016; Vilà et al., 2011). This means that in the long run the inherent characteristic attributed by the species and lack of their native competitors may experience a process of niche shift in new regions. Our prediction model has revealed that the area of high and moderate suitability for *P. juliflora* in future climate scenarios will increase relative to the current area. Within this context, further expansion *P. juliflora* might cause loss of biodiversity and reduce the cover of native herbaceous vegetation, which can further frustrate and affect pastoralist and agro-pastoralist livelihoods and societal well-being in Ethiopia in particular and in Africa in general. This is because climate change often favors invasive species as environmental conditions worsen for native species undermining their competitive power against invaders in ecosystem resources (Hellmann et al., 2008).

Most rangelands in Africa are located in arid and semi-arid ecosystem of the continent where *P. juliflora* has already invaded and will invade in the future as was predicted by the models of this study (Hoshino et al., 2012; Mbaabu et al., 2019; Shackleton et al., 2015; Shiferaw et al., 2019b). *P. juliflora* has been aggressively invading rangelands in many parts of Ethiopia particularly the Middle and Upper Awash Valley, Western and Eastern Harerge zones, and Afar and Somali regions (EBI, 2015). It has been documented that *P. juliflora* was one of factors threatening biodiversity and ecosystems in Eastern and Southern low lands of Ethiopia (EBI, 2015; Shiferaw et al., 2019c, Shiferaw et al., 2019d). This invasive alien species has been replacing more nutritive browsing vegetation, reducing the overall biodiversity of the areas; reducing the carrying capacities of rangelands, increasing incidence of crop pests and causing health problems (damaging eyes and hooves) of both domestic and wild animals, eventually leading to deaths (Ilukor et al., 2016). It was documented that invasion by *P. juliflora* have resulted in the loss of high quality and palatable plants in the Afar rangelands (EBI, 2015). The results of our study suggested that there would be a gain in both the highly and moderately suitable habitats particularly in the northern, north eastern and eastern pastoral areas of Ethiopia by the year 2050 and 2070. In the currently study we showed that climate change is strongly associated with the distribution of *P juliflora* in Ethiopia. Climate change is expected to become the major driver for the invasion of *P juliflora* and the loss of native species particularly grasses and herbaceous species in the future in synergy with the existing challenges and to contribute to the ongoing decline of grazing land in Ethiopia and other African countries at large. Range expansion of invasive species in the future can significantly reducing herd size due to reduced and impoverished watering and grazing points, and might be the major challenge the future of pastoralism and their livelihoods. It thus becomes imperative for stakeholders including but not limited to; scientific community, policy makers, land resource managers and other actors to refrain from the usual piecemeal approach and work together to develop efficient management strategies in order to prevent the expansion and also control and manage this invasive species in the country to reduce its negative impact on pastoral livelihood.

The invasion by *P. juliflora* reduced grass availability (feed availability) and carrying capacity of rangelands leading to overgrazing and land degradation (Ilukor et al., 2016; Shiferaw et al., 2019c, Shiferaw et al., 2019d). Sources of conflicts among different ethnic groups in pastoral areas include shortage of grazing land or need to access seasonally available forage and water. Expansion of *P. juliflora* directly contributes to such conflicts as it has been reducing area coverage and availability of rangelands (Shiferaw et al., 2019b). According to the perception of many pastoralists, livestock are everything for them (sources of food, income, security, social status, cultural value, insurance, etc.) and therefore, there are willing to pay any sacrifices to get forage and water for these animals (Bekele et al., 2018). This issue goes beyond biodiversity and economic reasons and has peace and security elements calling for integrated and coordinated actions at Federal and Regional levels to eradicating or minimizing the negative impacts of *P. juliflora* on pastoral production system (Rogers et al., 2017). According to Hundie and Padmanabhan (2008), the entire settlement farm was out of production and covered *P. juliflora* in Afar resulting in conversion of former rangeland into a land that was used for neither crop cultivation nor for livestock production. Haji and Mohammed (2013) reported that there was statistically significant negative effect of invasion by *P. juliflora* on the income from livestock and their products sale in Dire Dawa, Ethiopia. Invasion by this alien species might lead to decreased productivity of both crop and livestock which has direct implications for the worsening the livelihoods of the pastoralists.

## 5 Conclusion

Our result showed that *P. juliflora* is predicted to expand its distribution in different parts of the country. The continuous ranges expansion of the species has already caused adverse effects on biodiversity, ecosystem services and economy. The livelihood of many pastoralists in Africa in general and Ethiopia in particular depends on natural resources and associated ecosystem services for their survival. Our result showed that *P. juliflora* is predicted to expand aggressively to many drylands of parts of Ethiopia including covering significant lands in Afar, Oromia, Southern, Dire Dawa, Somalia, Amhara, Tigray and Gambella decreasing agricultural productivity and threatening local biodiversity. The current status and potential future increases in *P. juliflora* distribution and abundance in Ethiopia in particular and Africa in general call for coordinated and large scale interventions. Moreover, the results of the study will support management and early detection of invasive species in their potentially habitat suitable niches. Based on our study, we advise collaboration among different stakeholders for early identification and eradication actions at national level to design and implement comprehensive management strategies for *P. juliflora* that would eradicate or minimize the negative impacts.

## Declarations

### Author contribution statement

Dejene W. Sintayehu: Conceived and designed the experiments; Performed the experiments; Analyzed and interpreted the data; Contributed reagents, materials, analysis tools or data; Wrote the paper.

Gemedo Dalle, Arbo F. Bobasa: Conceived and designed the experiments; Analyzed and interpreted the data; Wrote the paper.

### Funding statement

This research did not receive any specific grant from funding agencies in the public, commercial, or not-for-profit sectors.

### Competing interest statement

The authors declare no conflict of interest.

### Additional information

No additional information is available for this paper.

## Appendix A Supplementary data

SI-Informations SI-Informations

## References

[bib1] Allouche O., Tsoar A., Kadmon R. (2006). Assessing the accuracy of species distribution models: prevalence, kappa and the true skill statistic (TSS). J. Appl. Ecol..

[bib2] Araújo M.B., New M. (2007). Ensemble forecasting of species distributions. Trends Ecol. Evol..

[bib3] Ayanu Y., Jentsch A., Müller-Mahn D., Rettberg S., Romankiewicz C., Koellner T. (2014). Ecosystem engineer unleashed: prosopis juliflora threatening ecosystem services?. Reg. Environ. Change.

[bib4] Beaury E.M., Fusco E.J., Jackson M.R., Laginhas B.B., Morelli T.L., Allen J.M., Pasquarella V.J., Bradley B.A. (2020). Incorporating climate change into invasive species management: insights from managers. Biol. Invasions.

[bib5] Bekele K., Haji J., Legesse B., Shiferaw H., Schaffner U. (2018). Impacts of woody invasive alien plant species on rural livelihood: Generalized propensity score evidence from Prosopis spp. invasion in Afar Region in Ethiopia. Pastoralism.

[bib6] Bellard C., Jeschke J.M., Leroy B., Mace G.M. (2018). Insights from modeling studies on how climate change affects invasive alien species geography. Ecol. Evol..

[bib7] Bradley B.A., Blumenthal D.M., Wilcove D.S., Ziska L.H. (2010). Predicting plant invasions in an era of global change. Trends Ecol. Evol..

[bib8] Conway D., Schipper L. (2011). Adaptation to climate change in Africa: challenges and opportunities identified from Ethiopia. Global Environ. Change.

[bib9] Corlett R.T., Westcott D.A. (2013). Will plant movements keep up with climate change ?. Trends Ecol. Evol..

[bib10] CSA (2016). Compendium of Environment Statistics, CSA.

[bib11] Diez J.M., D’Antonio C.M., Dukes J.S., Grosholz E.D., Olden J.D., Sorte C.J.B., Blumenthal D.M., Bradley B.A., Early R., Ibáñez I., Jones S.J., Lawler J.J., Miller L.P. (2012). Will extreme climatic events facilitate biological invasions?. Front. Ecol. Environ..

[bib12] EBI (Ethiopian Biodiversity Institute) (2015). Ethiopia's National Biodiversity Strategy and Action Plan 2015–2020.

[bib13] Eckert S., Hamad A., Kilawe C.J., Linders T.E.W., Ng W., Mbaabu P.R., Shiferaw H., Witt A., Schaffner U. (2020). Niche change analysis as a tool to inform management of two invasive species in Eastern Africa. Ecosphere.

[bib51] EEA (Ethiopian Economics Association) (2008). Climate Change and Development Adaptation Measures.

[bib14] Elith J.H., Graham C.P., Anderson R., Dudík M., Ferrier S., Guisan A.J., Hijmans R., Huettmann F.R., Leathwick J., Lehmann A., Li J., Lohmann G., Loiselle L.A., Manion B., Moritz G., Nakamura C., Nakazawa M., McC Y., Overton M., Townsend Peterson J., Phillips A.J., Richardson S., Scachetti-Pereira K., Schapire R.E., Soberón R., Williams J., Wisz S.S., Zimmermann E.N. (2006). Novel methods improve prediction of species’ distributions from occurrence data. Ecography (Cop.)..

[bib15] FAO (2006). Problems Posed By The Introduction of Prosopis spp.

[bib16] Felker P., Cadoret K., Harsh L.N., Cruz G., Tewari J.C., Maldonado L.J. (2001). The Prosopis Juliflora - Prosopis Pallida Complex: a Monograph.

[bib17] Getachew S., Demissew S., Woldemariam T. (2012). Allelopathic effects of the invasive Prosopis juliflora (Sw.) DC. on selected native plant species in mIddle Awash, Southern Afar Rift of Ethiopia. Manag. Biol. Invasions.

[bib18] Haji J., Mohammed A. (2013). Economic impact of prosopis juliflora on agropastoral households of Dire Dawa administration, Ethiopia. Afr. J. Agric. Res..

[bib19] Haregeweyn N., Tsunekawa A., Tsubo M. (2013). Analysis of the invasion rate, impacts and control measures of Prosopis juliflora: a case study of Amibara District, Eastern Ethiopia. Environ. Monit. Assess..

[bib20] Hayes M.A., Piaggio A.J. (2018). Assessing the potential impacts of a changing climate on the distribution of a rabies virus vector. PloS One.

[bib21] Hellmann J.J., Byers J.E., Bierwagen B.G., Dukes J.S. (2008). Five potential consequences of climate change for invasive species. Conserv. Biol..

[bib22] Heshmati I., Khorasani N., Shams-Esfandabad B., Riazi B. (2019). Forthcoming risk of Prosopis juliflora global invasion triggered by climate change: implications for environmental monitoring and risk assessment. Environ. Monit. Assess..

[bib23] Hoshino B., Karamalla A., Manayeva K., Yoda K., Suliman M., Elgamri M., Nawata H., Yasuda H. (2012). Evaluating the invasion strategic of mesquite (prosopis juliflora) in eastern Sudan using remotely sensed technique. J. Arid Land Stud..

[bib24] Hulme P.E. (2009). Trade, transport and trouble: managing invasive species pathways in an era of globalization. J. Appl. Ecol..

[bib25] Hundie B., Padmanabhan M. (2008). The transformation of the Afar commons in Ethiopia: state Coercion, diversification, and property rights change among pastoralists. CAPRi Working Paper.

[bib26] Ilukor J., Rettberg S., Treydte A., Birner R. (2016). To eradicate or not to eradicate ? Recommendations on Prosopis juliflora management in Afar , Ethiopia , from an interdisciplinary perspective. Pastoralism.

[bib27] Kariyawasam C.S., Kumar L. (2019). Invasive Plant Species Establishment and Range.

[bib28] Liu C., Berry P.M., Dawson T.P., Pearson R.G. (2005). Selecting thresholds of occurrence in the prediction of species distributions. Ecography (Cop.)..

[bib29] Mbaabu P.R., Ng W.T., Schaffner U., Gichaba M., Olago D., Choge S., Oriaso S., Eckert S. (2019). Spatial evolution of prosopis invasion and its effects on LULC and livelihoods in Baringo, Kenya. Rem. Sens..

[bib30] Mehari Z.H. (2015). The invasion of Prosopis juliflora and Afar pastoral livelihoods in the Middle Awash area of Ethiopia. Ecol. Process..

[bib31] Mohammed M., Abdulahi J.A.U., Regasa T. (2018). Prosopis Juliflora l : distribution , impacts and available control methods in Ethiopia. Trop. Subtrop. Agroecosystems.

[bib32] Phillips J. Steven, Robert P., Anderson R.E.S. (2006). Maximum entropy modeling of species geographic distributions. Ecol. Model..

[bib33] Qin Z., Zhang J.E., DiTommaso A., Wang R.L., Liang K.M. (2016). Predicting the potential distribution of Lantana camara L. under RCP scenarios using ISI-MIP models. Clim. Change.

[bib34] R Core Team (2017). Species distribution modeling with R Introduction. R Proj. Stat. Comput..

[bib35] Ren Z., Wang D., Ma A., Hwang J., Bennett A. (2020). Predicting malaria vector distribution under climate change scenarios in China : challenges for malaria elimination. Nat. Publ. Gr..

[bib36] Rogers P., Nunan F., Fentie A.A. (2017). Reimagining invasions: the social and cultural impacts of Prosopis on pastoralists in southern Afar, Ethiopia. Pastoralism.

[bib37] Shackleton R.T., Le Maitre D.C., Richardson D.M. (2015). Prosopis invasions in South Africa: population structures and impacts on native tree population stability. J. Arid Environ..

[bib38] Shiferaw W., Demissew S., Bekele T. (2018). Invasive alien plant species in Ethiopia: ecological impacts on biodiversity a review paper. Int. J. Mol. Biol..

[bib39] Shiferaw H., Bewket W., Eckert S. (2019). Performances of machine learning algorithms for mapping fractional cover of an invasive plant species in a dryland ecosystem. Ecol. Evol..

[bib40] Shiferaw, Bekele T., Demissew S., Aynekulu E. (2019). Prosopis juliflora invasion and environmental factors on density of soil seed bank in Afar Region, Northeast Ethiopia. J. Ecol. Environ..

[bib41] Shiferaw H., Bewket W., Alamirew T., Zeleke G., Teketay D., Bekele K. (2019). Implications of land use/land cover dynamics and Prosopis invasion on ecosystem service values in Afar Region , Ethiopia. Sci. Total Environ..

[bib42] Shiferaw H., Scha U., Bew W., Alamirew T., Zele G. (2019). Modelling the Current Fractional Cover of an Invasive Alien Plant and Drivers of its Invasion in a Dryland Ecosystem.

[bib43] Shrestha U.B., Sharma K.P., Devkota A., Siwakoti M., Shrestha B.B. (2018). Potential impact of climate change on the distribution of six invasive alien plants in Nepal. Ecol. Indicat..

[bib44] Simberloff D., Martin J.L., Genovesi P., Maris V., Wardle D.A., Aronson J., Courchamp F., Galil B., García-Berthou E., Pascal M., Pyšek P., Sousa R., Tabacchi E., Vilà M. (2013). Impacts of biological invasions: what’s what and the way forward. Trends Ecol. Evol..

[bib45] Sintayehu D.W. (2018). Impact of climate change on biodiversity and associated key ecosystem services in Africa: a systematic review. Ecosyst. Heal. Sustain..

[bib46] Thomas C.D., Cameron A., Green R.E., Bakkenes M., Beaumont L.J., Collingham Y.C., Erasmus B.F.N., Ferreira De Siqueira M., Grainger A., Hannah L., Hughes L., Huntley B., Van Jaarsveld A.S., Midgley G.F., Miles L., Ortega-Huerta M.A., Peterson A.T., Phillips O.L., Williams S.E. (2004). Extinction risk from climate change. Nature.

[bib47] Vilà M., Espinar J.L., Hejda M., Hulme P.E., Jarošík V., Maron J.L., Pergl J., Schaffner U., Sun Y., Pyšek P. (2011). Ecological impacts of invasive alien plants: a meta-analysis of their effects on species, communities and ecosystems. Ecol. Lett..

[bib48] Wakie T.T., Evangelista P.H., Jarnevich C.S., Laituri M. (2014). Mapping current and potential distribution of non-native prosopis juliflorain the Afar region of Ethiopia. PloS One.

[bib49] Witt A., Beale T., Van Wilgen B.W., Witt A., Beale T., Van Wilgen B.W., Witt A., Beale T., Van Wilgen B.W. (2018). Transactions of the Royal Society of South Africa an assessment of the distribution and potential ecological impacts of invasive alien plant species in eastern Africa an assessment of the distribution and potential ecological impacts of invasive alien pla. Trans. R. Soc. South Africa.

[bib50] Zeray N., Legesse B., Mohamed J.H., Aredo M.K. (2017). Impacts of Prosopis juliflora invasion on livelihoods of pastoral and agro-pastoral households of Dire Dawa Administration. Pastoralism.

