# Supplementary material for: Impacts of climate change on current and future invasion of Prosopis juliflora in Ethiopia: environmental and socio-economic implications
Source: Heliyon. 2020 Aug 1;6(8):e04596. doi: 10.1016/j.heliyon.2020.e04596 (PMC7398938; doi:10.1016/j.heliyon.2020.e04596)
Supplement: SI-Informations [file mmc1.doc]

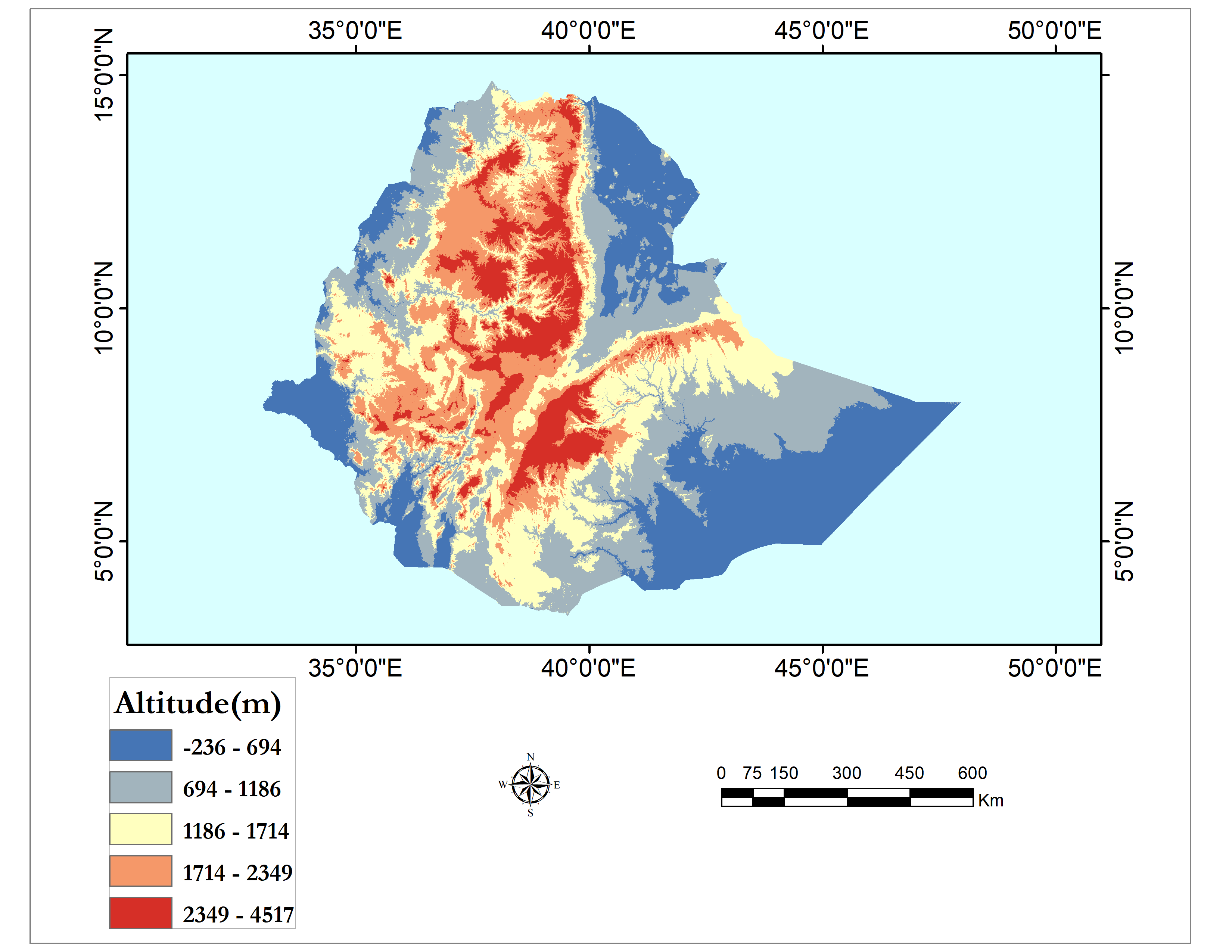


SI-Figure 1: Elevation map of Ethiopia


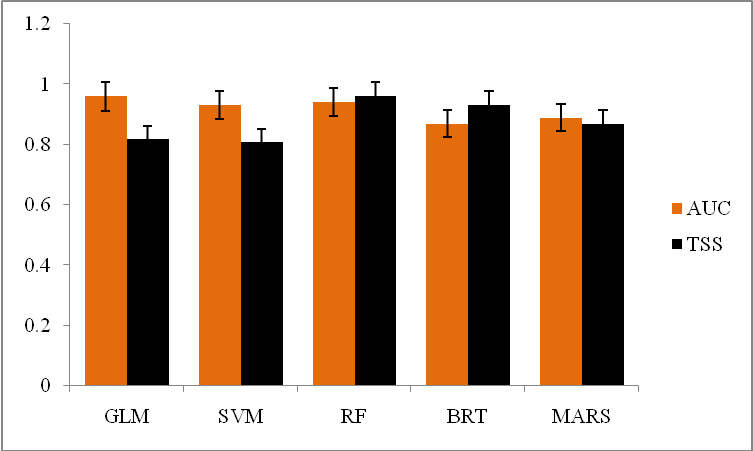


SI-Figure 2. AUC and TSS mean values of five models used for predicting *P. juliflora* distribution in Ethiopia.


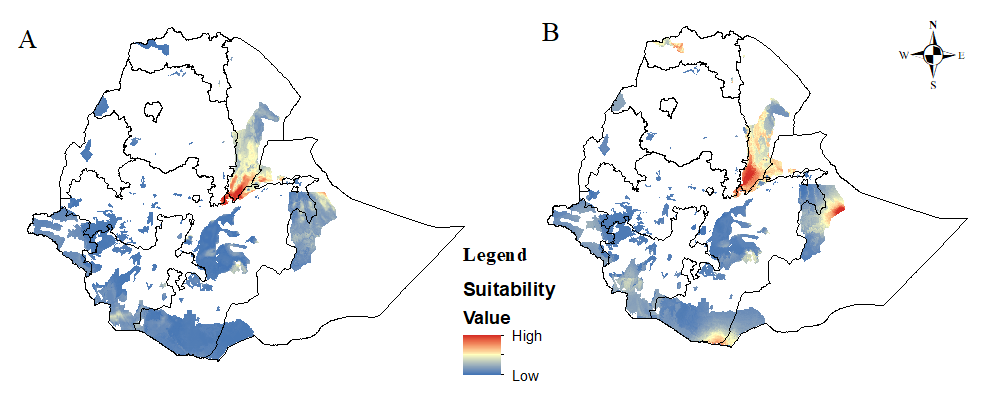


SI-Figure 3. Current (A) and future (B) habitat suitability for *P. juliflora* in protected areas of Ethiopia. Blue to red colours illustrate gradients of habitat suitability from low to high

SI-Table 1. Predictor variables used for modelling the potential distribution of *P. juliflora*.

| Label | Variable | Units |
| --- | --- | --- |
| bio1 | Annual mean temperature | Degree Celsius |
| bio2 | Diurnal range (Mean of monthly (max temp - min temp)) | Degree Celsius |
| bio9 | Mean temperature of driest quarter | Degree Celsius |
| bio12 | Annual precipitation | Millimeter |
| bio14 | Precipitation of driest month | Millimeter |
| bio15 | Precipitation seasonality (Coefficient of Variation) | Percentage |
| LC | Land cover |  |
